# Effect of combined periodontal orthodontic treatment in periodontal subjects having anterior displaced teeth

**DOI:** 10.6026/973206300223175

**Published:** 2026-05-31

**Authors:** Padma Panni, Manish Dagdiya, Subhash Chandra Nayak, Akshay Bansal, Awanindra Kumar Jha, Lipsa Behera

**Affiliations:** 1 Department of Orthodontics and Dentofacial Orthopaedics, Shree Krishna Medical College and Hospital, Muzaffarpur, Bihar, India; 2 Department of Orthodontics and Dentofacial Orthopaedics, Saraswati Dhanvantri Dental College, Parbhani, Maharashtra, India; 3 Department of Orthodontics and Dentofacial Orthopaedics, Hi-Tech Dental College and Hospital, Pandara Bhubaneswar, Odisha, India;; 4 Department of Periodontics, Inderprastha Dental College and Hospital, Shahibabad, Ghaziabad, Uttar Pradesh, India; 5 Department of Orthodontics and Dentofacial Orthopaedics, Dental Institute, Rajendra Institute of Medical Sciences Bariatu, Ranchi, Jharkhand, India

**Keywords:** Anteriorly displaced teeth, bone defects, fibrotomy, periodontal-orthodontic treatment

## Abstract

Periodontitis with anteriorly displaced incisors results in poor dental functioning and esthetics. Therefore, it is of interest to assess the efficacy of combined periodontal orthodontic treatment in periodontal subjects having anterior displaced teeth. Using circumferential supracrestal fibrotomy, orthodontic-periodontal therapy was performed on 28 individuals who had 56 enlarged maxillary incisors with horizontal bone abnormalities. GTR (guided tissue regeneration) was used in periodontal regenerative surgery to improve bone morphology in anterior teeth with angular bone deficiencies after orthodontic therapy. According to the study findings, bone redeposition was observed radiographically with p=0.05 and clinical attachment loss and probing depth significantly improved from T0 to T1. Periodontal problems in bone defects can be improved with combined orthodontic and periodontal therapy. Thus, Fibrotomy and orthodontic intrusion alter bone shape, which may enhance further directed tissue regeneration.

## Background:

Periodontitis is a common clinical condition in subjects visiting dental care and periodontal diseases result in the loss of supporting structures of teeth. When the structures supporting the teeth maintain the physiological position of the teeth and are disturbed by periodontal disease, subjects can present pathologic tooth migration including drifting, extrusion, rotation, diastema and/or proclination. Periodontitis with anteriorly displaced incisors results in acceleration of loss of periodontal supporting structures and premature occlusion contact that negatively affects dental esthetics. In treating these subjects, orthodontic treatment is usually needed [1]. Periodontal regeneration can be considered as de novo osteogenesis, cement genesis and newly formed fibres regeneration in both newly formed alveolar bone and cementum. With appropriate orthodontic-periodontal coordination, it is vital to reestablish a functional and healthy dentition [2]. Orthodontic tooth movement is a process of alveolar bone remodeling with occlusive reconstruction and changes in bone morphology. The bone loss during teeth intrusion including periodontal ligament space enlargement at a cervical area or periodontal pocket deepening is attributed to junctional epithelium displacement that will need marginal bone repositioning for preservation of periodontal width [3].

Circumferential supracrestal fibrotomy therapy usually reduces rotated teeth relapse. Recently, it has been reported that orthodontic intrusion with circumferential supracrestal fibrotomy is an efficacious management for anterior teeth extrusion. Circumferential supracrestal fibrotomy surgery controls marginal bone resorption following tooth intrusion and reduces marginal bone loss. It can lead to cone-shaped defect creation with intrusion; hence, the horizontal bone defect can be altered to angular [4]. In a clinical context, periodontitis subjects usually have alveolar bone loss depicting 2 patterns vertical (angular) and horizontal defects. In angular bone loss, bone resorption for one tooth-sharing septum is greater than for another tooth. Infrabony pockets are linked to angular bone loss. Horizontal bone loss is a more common type of alveolar bone resorption. Bone is equally lost from 2 adjacent teeth with the flat interproximal bone level being flat and the deepest pocket portion located coronal to the alveolar crest. It is still controversial whether orthodontic treatment can help in attaining new attachments [4]. Both grafting procedure and guided tissue regeneration are means of attaining periodontal regeneration. However, elongated incisors and flaring usually lead to horizontal bone defects that are not suitable for periodontal regenerative procedures. If bone defect morphology can be altered from horizontal to angular by orthodontic treatment, guided tissue regeneration will increase long-term stability and periodontal tissue improvement [6]. Therefore, it is of interest to show the efficacy of combined periodontal orthodontic treatment in periodontal subjects having anterior displaced teeth.

## Materials and Methods:

The present clinical study aimed to assess the efficacy of combined periodontal orthodontic treatment in periodontal subjects having anterior displaced teeth. The study was done at Department of Orthodontics & Dentofacial Orthopaedics, Saraswati Dhanvantri Dental College, Parbhani, Maharashtra, after the clearance was given by the concerned Institutional Ethical committee. Verbal and written informed consent was taken from all the subjects before participation. The study assessed 28 subjects diagnosed with chronic periodontitis with 22 females and 6 males aged 22-41 years. The inclusion criteria for the study were subjects who needed orthodontic treatment, panoramic radiographs depicting horizontally shaped bone defects, extrusion and migration of maxillary anterior teeth from periodontal diseases, good oral hygiene with full-mouth bleeding and plaque scores as <25% and <15% respectively, periodontal disease earlier managed by scaling and oral hygiene instructions, non-smoking status, good general and systemic health and no systemic disease. The treatment procedure was initial periodontal therapy comprising of scaling, root planning and oral hygiene instructions before orthodontic treatment. Subjects were managed periodontally till resolution of inflammation via control of bacterial biofilm and oral hygiene maintenance with full-mouth bleeding and plaque scores as <25% and <15% respectively. Conventional circumferential supracrestal fibrotomy technique was done by a single expert periodontist during incisor intrusion once every month and severed epithelial attachment around the teeth and transected the transseptal fibers by entering periodontal ligament space interdentally. Root planning was done at the same time and root planing was done to prevent inflammatory granulation tissue formation around the periodontal space. In orthodontic treatment, fixed appliances were placed on the first molars and maxillary incisors for anchorage. Subjects were managed with intrusive mechanisms using titanium alloy wire to realign and intrusion of migrated incisors and for space closure. Appliances were immediately loaded after the first circumferential supracrestal fibrotomy. Light forces of 10-15g per tooth were used based on residual periodontal support. Following intrusion, teeth other than first molars and incisors were bonded for leveling and alignment of the arch. Appliances were adjusted every 4 weeks and prophylaxis was done every 3 months. Active orthodontic treatment was continued for a mean of 19 months. Fixed appliances were placed in the mouth till guided tissue regeneration. Subjects with angular bone defects around maxillary incisors were given GTR surgery by the same periodontist. After completing treatment, all subjects were given resin-bonded splints on lingual anterior teeth for retention. For evaluation of alveolar bone changes during maxillary incisor retraction, CBCT (cone-beam computed tomography) images were taken at T0, T1 (after orthodontic treatment) and T2 (after 6 months of GTR). To reduce radiation, maxillary incisors were included in a single image. CBCT was done for treatment. For GTR, detailed information was gathered concerning defect shape. CBCT images provided better quantitative and diagnostic information compared to conventional radiography on periodontal bone levels in three dimensions. An optimal visualization of teeth was done in sagittal, coronal and axial planes. For periodontal examination at T0 and T1, clinical attachment loss and probing pocket depth were assessed in millimetres using William's probe on each maxillary incisor. At T1 and T2, clinical attachment loss and probing pocket depth were assessed on teeth that received GTR. To measure bone morphology from T0 to T1, measurements taken were marginal bone loss as distance from the marginal bone crest to the cementoenamel junction. Palatal and labial bone levels were measured in sagittal planes and distal and mesial bone levels were in coronal plane. Total, palatal and labial alveolar bone thickness was assessed in the sagittal plane at the apical level. If the horizontal bone defect changed to a narrow-deep defect following orthodontic intrusion, the bone defect angle was measured and defined by 2 lines where the first line was between the bottom of the bone defect and the most coronal extension of the interproximal bone crest and the second line was the root surface. Changes in infrabony defects from T1 to T2 following GTR were assessed. The vertical distance between the projection of the alveolar crest on the root surface and the most apical point of the bone defect, as well as the horizontal distance from the marginal bone crest and the root surface, were assessed. In infrabony defect, marginal bone loss was assessed as the distance from the most apical point of the bone defect to the cementoenamel junction. For both T0-T1 and T1-T2 stages, radiographs were assessed twice. Measurements were done at 2-week intervals. A mean of two measurements was taken. The data gathered were analyzed statistically using SPSS (Statistical Package for the Social Sciences) software version 24.0 (IBM Corp., Armonk. NY, USA) for assessment of descriptive measures, Student t-test, ANOVA (analysis of variance) and Chi-square test. The results were expressed as mean and standard deviation and frequency and percentages. The p-value of <0.05 was considered.

## Results:

The present clinical study aimed to assess the efficacy of combined periodontal orthodontic treatment in periodontal subjects having anterior displaced teeth. The study assessed 28 adults with 56 elongated maxillary incisors having horizontal bone defects that underwent orthodontic-periodontal treatment using circumferential supracrestal fibrotomy. It was seen that the mean decrease in CAL value was significant from T0 to T1 with p=0.03. However, mean PPD reduction was statistically non-significant from T0 to T1 with p=0.302. Change in labial and palatal plate thickness from To-T1 was statistically significant with p=0.002 and 0.02. However, combined labial and palatal plate thickness showed a non-significant difference from T0-T1 with p=0.57. The decrease in palatal, labial, distal and mesial defects from To-T1 was statistically significant with p=0.006, 0.001, <0.001 and <0.001 respectively. Mean bone defect reduction from T0-T1 was also statistically significant with p<0.001 (Table 1). The study results also showed that concerning bone morphology, there were 34 angular bone defects in the present study seen in 28 teeth of 16 subjects after completion of the orthodontic treatment. The angle for 8 labial bone defects was 15.77, 15.79, 29.68, 29.70 and 15.59. 15.61, 18.14 and 18.16 respectively. The bone defect angle for 12 mesial bone defects was 27.49. 27.51, 36.36, 36.38, 26.86, 26.88, 34.36, 34.38, 31.87, 31.89, 25.09 and 25.11. The bone defect angles for 14 distal bone defects were 29.85, 29.87, 20.11, 20.13, 20.68, 20.70, 31.73, 31.75, 30.91, 30.93, 39.72, 39.74, 24.78 and 24.80 degrees respectively. It was also seen that for assessing the alterations in infrabony defects in study subjects at T1 and T2, the mean reduction in clinical attachment level and pocket probing depth was statistically significant from T1 to T2 i.e., after orthodontic treatment and 6 months after GTR with p=0.000 and 0.000. MBC-TD (marginal bone crest to the projection of the alveolar crest on the root surface) showed a significant increase from T1 to T2 with p=0.001. TD-ABD (projection of the alveolar crest on the root surface- most apical point of the bone defect) showed a significant decrease from T1 to T2 with p=0.000. CEJ-ABD also showed a significant decrease from T1 to T2 with p=0.005 (Table 2).

## Discussion:

The present study assessed 28 adults with 56 elongated maxillary incisors having horizontal bone defects who underwent orthodontic-periodontal treatment using circumferential supracrestal fibrotomy. It was seen that the mean decrease in CAL value was significant from T0 to T1 with p=0.03. However, mean PPD reduction was statistically non-significant from T0 to T1 with p=0.302. Change in labial and palatal plate thickness from To-T1 was statistically significant with p=0.002 and 0.02. However, combined labial and palatal plate thickness showed a non-significant difference from T0-T1 with p=0.57. The decrease in palatal, labial, distal and mesial defects from To-T1 was statistically significant with p=0.006, 0.001, <0.001 and <0.001 respectively. Mean bone defect reduction from T0-T1 was also statistically significant with p<0.001. These results were consistent with the studies of Jiang *et al.* [7] in 2025 and Liu *et al.* [8] in 2023 where authors assessed subjects with anteriorly displaced teeth undergoing orthodontic treatment and had periodontal defects and similar CAL, PPD and palate thickness change similar to the present study was also reported by the authors in their respective studies. The study results showed that concerning bone morphology, there were 34 angular bone defects in the present study seen in 28 teeth of 16 subjects after completion of the orthodontic treatment. The angle for 8 labial bone defects was 15.77, 15.79, 29.68, 29.70 and 15.59. 15.61, 18.14 and 18.16 respectively. The bone defect angle for 12 mesial bone defects was 27.49. 27.51, 36.36, 36.38, 26.86, 26.88, 34.36, 34.38, 31.87, 31.89, 25.09 and 25.11. The bone defect angles for 14 distal bone defects were 29.85, 29.87, 20.11, 20.13, 20.68, 20.70, 31.73, 31.75, 30.91, 30.93, 39.72, 39.74, 24.78 and 24.80 degrees respectively. These findings were in agreement with the results of Huang *et al.* [9] in 2021 and Rekhawat *et al.* [10] in 2020 where bone morphology parameters reported by the authors in their studies were comparable to the results of the present study. It was seen that for assessing the alterations in infrabony defects in study subjects at T1 and T2, the mean reduction in clinical attachment level and pocket probing depth was statistically significant from T1 to T2 i.e., after orthodontic treatment and 6 months after GTR with p=0.000 and 0.000. MBC-TD (marginal bone crest to the projection of the alveolar crest on the root surface) showed a significant increase from T1 to T2 with p=0.001. TD-ABD (projection of the alveolar crest on the root surface- most apical point of the bone defect) showed a significant decrease from T1 to T2 with p=0.000. CEJ-ABD also showed a significant decrease from T1 to T2 with p=0.005. These results were in line with the findings of Ghouraba *et al.* [11] in 2024 and Celis *et al.* [12] in 2025 where alterations in infrabony defects after GTR and post-orthodontic treatment comparable to the present study were also reported by the authors in their respective studies.

## Conclusion:

Combined orthodontic-periodontal treatment helps in improving the periodontal conditions in the defective bone sites. Bone morphology is changed by the orthodontic intrusion with fibrotomy which can help in the improvement of further guided tissue regeneration. However, the study had limitations of a smaller sample size and shorter follow-up. Hence, further longitudinal studies with larger sample sizes and linger monitoring are needed to reach a definitive conclusion.

## Figures and Tables

**Table 1:** Changes in alveolar bone periodontal parameters in study subjects at T0 and T1

**S. No**	**Measurement (mm)**	**Mean T0**	**Mean T1**	**T1-T0**	**p-value**
1	CAL	3.37±1.45	3.08±1.14	-0.27±0.15	0.03
2	PPD	2.73±0.90	2.66±1.78	-0.05±0.73	0.302
3	Labial plate thickness	1.8±1.06	2.57±1.52	0.52±1.00	0.002
4	Palatal plate thickness	5.65±1.63	5.19±1.79	-0.44±1.20	0.02
5	Labial+ palatal plate thickness	7.70±1.36	7.79±1.51	0.06±0.89	0.57
6	Palatal	4.24±1.83	3.66±1.70	-0.56±1.17	0.006
7	Labial	4.60±1.18	4.071.33	-0.51±0.96	0.001
8	Distal	5.40±1.68	4.67±1.43	-0.71±0.82	<0.001
9	Mesial	5.03±1.59	4.26±1.23	-0.74±1.12	<0.001
10	Mean	4.84±1.63	4.19±1.46	-0.64±1.02	<0.001

**Table 2:** Alterations in infrabony defects in study subjects at T1 and T2

**S. No**	**Measurement (mm)**	**Mean T1**	**Mean T2**	**T2-T1**	**p-value**
1	CAL	7.00±1.79	3.81±1.53	-3.28±1.55	0
2	PPD	5.89±1.03	3.00±1.07	-2.87±1.14	0
3	MBC-TD	1.54±0.61	2.09±0.58	-1.42±0.90	0.001
4	TD-ABD	4.26±0.87	2.10±1.25	-2.13±0.66	0
5	CEJ-ABD	6.26±0.83	4.15±1.16	-2.09±1.28	0.005

## References

[1] https://www.ncbi.nlm.nih.gov/books/NBK541126/.

[2] Huang TH (2024). Biomedicines..

[3] Jeon HH (2021). J Clin Med..

[4] Song BJ (2022). Applied Sciences..

[5] Bud E (2025). Dent J (Basel)..

[6] Antonarakis GS (2000). Periodontology.

[7] Jiang B (2025). Am J Transl Res..

[8] Liu Y (2023). Pak J Med Sci..

[9] Huang YZ (2021). Medicine..

[10] Ghouraba A (2020). Turk J Orthod..

[11] Huang RF (2024). BMC Oral Health..

[12] Celis B (2025). J Clin Periodontol..

